# MIRO and IRbase: IT Tools for the Epidemiological Monitoring of Insecticide Resistance in Mosquito Disease Vectors

**DOI:** 10.1371/journal.pntd.0000465

**Published:** 2009-06-23

**Authors:** Emmanuel Dialynas, Pantelis Topalis, John Vontas, Christos Louis

**Affiliations:** 1 Institute of Molecular Biology and Biotechnology, Foundation for Research and Technology-Hellas, Heraklion, Crete, Greece; 2 Laboratory of Pesticide Science, Agricultural University of Athens, Athens, Greece; 3 Vector Research, Liverpool School of Tropical Medicine, Liverpool, United Kingdom; 4 Department of Biology, University of Crete, Heraklion, Crete, Greece; Mahidol University, Thailand

## Abstract

**Background:**

Monitoring of insect vector populations with respect to their susceptibility to one or more insecticides is a crucial element of the strategies used for the control of arthropod-borne diseases. This management task can nowadays be achieved more efficiently when assisted by IT (Information Technology) tools, ranging from modern integrated databases to GIS (Geographic Information System). Here we describe an application ontology that we developed *de novo*, and a specially designed database that, based on this ontology, can be used for the purpose of controlling mosquitoes and, thus, the diseases that they transmit.

**Methodology/Principal Findings:**

The ontology, named MIRO for Mosquito Insecticide Resistance Ontology, developed using the OBO-Edit software, describes all pertinent aspects of insecticide resistance, including specific methodology and mode of action. MIRO, then, forms the basis for the design and development of a dedicated database, IRbase, constructed using open source software, which can be used to retrieve data on mosquito populations in a temporally and spatially separate way, as well as to map the output using a Google Earth interface. The dependency of the database on the MIRO allows for a rational and efficient hierarchical search possibility.

**Conclusions/Significance:**

The fact that the MIRO complies with the rules set forward by the OBO (Open Biomedical Ontologies) Foundry introduces cross-referencing with other biomedical ontologies and, thus, both MIRO and IRbase are suitable as parts of future comprehensive surveillance tools and decision support systems that will be used for the control of vector-borne diseases. MIRO is downloadable from and IRbase is accessible at VectorBase, the NIAID-sponsored open access database for arthropod vectors of disease.

## Author Summary

It is a historical fact that a successful campaign against vector populations is one of the prerequisites for effectively fighting and eventually eradicating arthropod-borne diseases, be that in an epidemic or, even more so, in endemic cases. Based mostly on the use of insecticides and environmental management, vector control is now increasingly hampered by the occurrence of insecticide resistance that manifests itself, and spreads rapidly, briefly after the introduction of a (novel) chemical substance. We make use here of a specially built ontology, MIRO, to drive a new database, IRbase, dedicated to storing data on the occurrence of insecticide resistance in mosquito populations worldwide. The ontological approach to the design of databases offers the great advantage that these can be searched in an efficient way. Moreover, it also provides for an increased interoperability of present and future epidemiological tools. IRbase is now being populated by both older data from the literature and data recently collected from field.

## Introduction

Diseases transmitted by arthropod vectors and, in particular, mosquitoes pose an immense load on global health, with malaria alone being responsible for more than 46,000,000 DALYs (Disease-adjusted Life Years); pertinent calculations are based solely on official, yet largely incomplete statistical estimates [1], and the global burden of *falciparum* malaria is nowadays estimated by some to be lower than originally thought [2]. Nevertheless, given the fact that arthropod-borne diseases affect mostly the populations of tropical regions, these huge numbers directly imply that their control is a *conditio sine qua non* for the socio-economic development of many of the poor areas of the world. Control of disease, then, directly entails the control of the arthropod vector populations and, most prominently among them, mosquitoes. Of course, economic development itself is one of the key players in the control of vector-borne diseases, unfortunately leading to an argument of a spiral form [3]. However, since the original recognition of the causes of malaria and other tropical diseases, campaigns aiming at eradicating vector-borne diseases included environmental management [4], indoor residual spraying (IRS) with the widespread use of DDT (Dichloro-Diphenyl-Trichloroethane) or other insecticides [5],[6], as well as the use of impregnated nets (Insecticide-Treated Nets, ITN [7]; and Long-Lasting Insecticide-treated Nets, LLIN [8]). These approaches, combined with extensive use of drugs, soon led to the disappearance of the disease from most non-tropical areas of the world and notably Europe [9].

In spite of the initial wide successes achieved in the temperate zones, eradication of vector-borne diseases proved to be elusive in the tropics. Moreover, the failure of vaccine development for vector-borne diseases, with the exception of the relatively early production of a vaccine directed against yellow fever [10], complicated the strategies aimed at controlling these diseases. Perhaps, most prominent among several problems that were faced by the national and international public health agencies were the occurrences of resistance relating to both parasites becoming resistant to anti-parasitic drugs [11] and mosquitoes to insecticides [12]. The gradual development of insecticide resistance against all classes of insecticides used today soon after their introduction [13], which was exacerbated by the use of such chemicals in agriculture [14], is considered by some to be presently the most important impediment in the successful control of vector-borne diseases.

Resistance to one or more insecticides used in vector control can have a crucial impact on the management of arthropod vector-borne diseases. In the case of ITN and LLIN measures [15],[16], monitoring of insecticide resistance needs to become a key component for the efficient usage of control strategies [17]. Although overall data on pesticide resistance have been collected over a long period of time [18]–[20], these often remain inaccessible to public health workers around the world for a variety of reasons. One of them is the lack of a central database tool that would gather, store and exploit such data. Although pertinent studies are often published in refereed journals, their accessibility is limited by the use of restrictions, such as expensive subscription, something that is of extreme importance to scientists from disease-endemic countries, namely the very ones who urgently need to access these data.

With this in mind we decided to develop IT tools that could offer solutions to some of the problems and most importantly to help monitor the occurrence of insecticide resistance in vector populations; we decided to first focus on mosquitoes as these represent the most important vectors of disease. Rather than only expanding the simple repository of insecticide resistance studies that we had previously developed [21], we decided to completely restructure the database and support it by a dedicated ontology (or controlled vocabulary). This type of tool, which among others helps standardize terminology in a computer-comprehensible form, has already proved its immense potential in cases such as, most prominently, the Gene Ontology (GO) project [22]. Both the ontology (hereafter called MIRO for Mosquito Insecticide Resistance Ontology) and the novel, enhanced database (called IRbase) are freely accessible to the research community through their incorporation in VectorBase [23],[24].

## Materials and Methods

A Dell PowerEdge 850, with a dual core Intel Pentium D CPU running at 3 GHz, 3 GB of RAM, and 150 GB of hard disk storage was used for the development of IRbase. The operating system used is CentOS 4.5 and the web service is handled by the Apache server. Both MySQL and PostgresQL database servers were used for data storage. Webpage scripts and command line scripts are written in PHP. For PHP development we have been using the Zend Development Environment (ZDE). The OBO-edit software package [25] was used for the development of the MIRO.

To display the locations of the collection sites the Google Maps API and maps are used. Geographic data are exchanged between the applications using the Keyhole Markup Language (KML), a data schema for annotating and visualizing two or three dimensional maps. All coordinates are based on the World Geodetic System (WGS) 84 projection standard.

Data are entered through the online AJAX web interface, which is ontology based. Alternatively, submitters may send in their data in Open Office (ods), Excel (xls), comma separated values (csv), or tab separated values (tsv) files, which are processed and imported into the database using PHP scripts.

The MIRO can be accessed and browsed at the URL http://www.vectorbase.org/Search/CVSearch/ and its latest version can be downloaded from http://anobase.vectorbase.org/miro/miro_release.obo; it is also available through the OBO-Foundry at http://obofoundry.org/cgi-bin/detail.cgi?mosquito_insecticide_resistance; the home page for the IRbase is at the URL http://anobase.vectorbase.org/ir/. Both MIRO and IRbase are freely accessible. To access all necessary files for a local usage of IRbase the authors should be contacted by e-mail (louis@imbb.forth.gr).

## Results/Discussion

### The MIRO ontology

For the construction of the MIRO we followed the rules established by the OBO Foundry [26] in order to establish maximum interoperability in the future. This implied the use, to some extent, of already established ontologies, rather than the *de novo* development of new ones, such as the geographical component (see below). This decision obviously restricted the usage of relations linking terms to those allowed by the OBO Foundry rules and thus only *is_a*, *part_of* and *agent_in* are used throughout [27]. We are convinced, though, that this choice increases cross-ontology coordination and makes the tools developed more amenable to integration in a suite of malaria decision tools that are being developed.

The next choice we were faced with was the one of whether this ontology should follow the ontological scaffold and the rules and conventions described for the Basic Formal Ontology [28]. This ontological arrangement is already used for a variety of biomedical ontologies, including anatomical ontologies of disease vectors, notably mosquitoes and ticks [29]. Although the obvious advantages of a BFO-based ontology such as, for example, the ease of expansion that is based on its modularity cannot be easily discarded, we decided to initially design the MIRO on a more “traditional” scheme that would make it easily recognizable by users who are not proficient in ontologies. The single reason for this is to be able to provide the insecticide resistance community with a module that can be easily incorporated into other IT tools currently being devised. Nevertheless we are in the process of transporting the MIRO into a BFO-based format in order to be able to integrate that version in future constructs that would potentially require such a layout.

**Figure 1:**
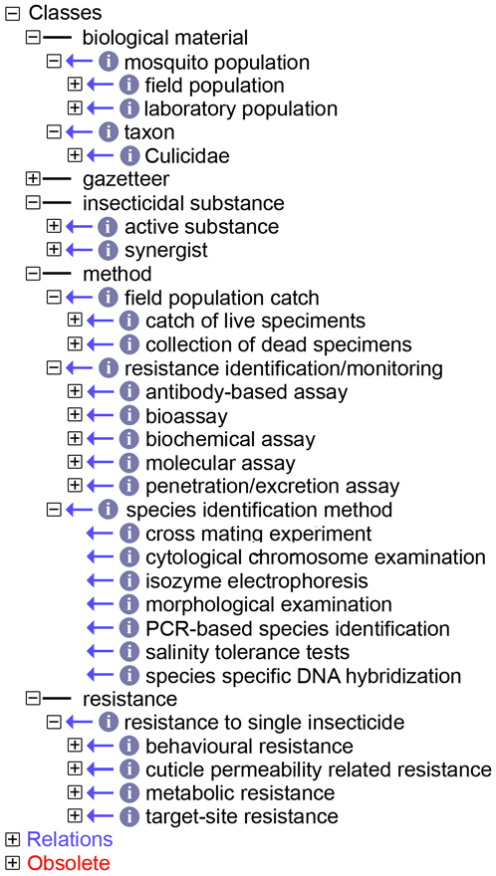
The upper levels of the MIRO. The figure shows the upper levels of the ontology. The small circles denote an “*is_a*” relation between the term and its parent, and small rectangles show the presence (plus) or absence (minus) of children for a given term. The children of the four “*biological material*”, “*insecticidal substance*”, “*method*” and “*resistance*” are shown in a depth of 2 levels. The “*gazetteer*” class has been loaded into MIRO (see Results and Discussion) and is therefore visible here.

MIRO is based on five top-level classes that actually form independent sub-ontologies (see Figure 1); four of them, “*biological material*”, “*insecticidal substance*”, “*method*” and “*resistance*”, were developed *de novo* by us explicitly for the MIRO. In Figure 1 (left part) the ontology's terms are shown in a depth of two levels with the exception of the fifth class, the “*gazetteer*”. This class represents a full importation of the Gazetteer (GAZ), a controlled vocabulary following ontological rules that describes named geographical locations (http://darwin.nerc-oxford.ac.uk/gc_wiki/index.php/GAZ_Project). GAZ is a community-based project of the EnvO Consortium for describing instances of organism environments and biological samples, supporting consistent annotation of locations and environments. The Gazetteer describes places and place names and the relations between them. Here, GAZ is basically used to describe the locations of sampling. Although it is a fully integrated component of MIRO, due to its size GAZ is not incorporated as such in our ontology, but it is automatically loaded through the Internet every time that one opens the MIRO using the OBOedit software. At this moment the MIRO contains 4,291 terms excluding, of course, the GAZ component that contains more than one hundred and fifty thousand geographical names from all over the world; more than 99% of the MIRO terms have full definitions. It should be noted that terms are not fixed and more are being added as these become necessary.

#### Biological material

**Figure 2:**
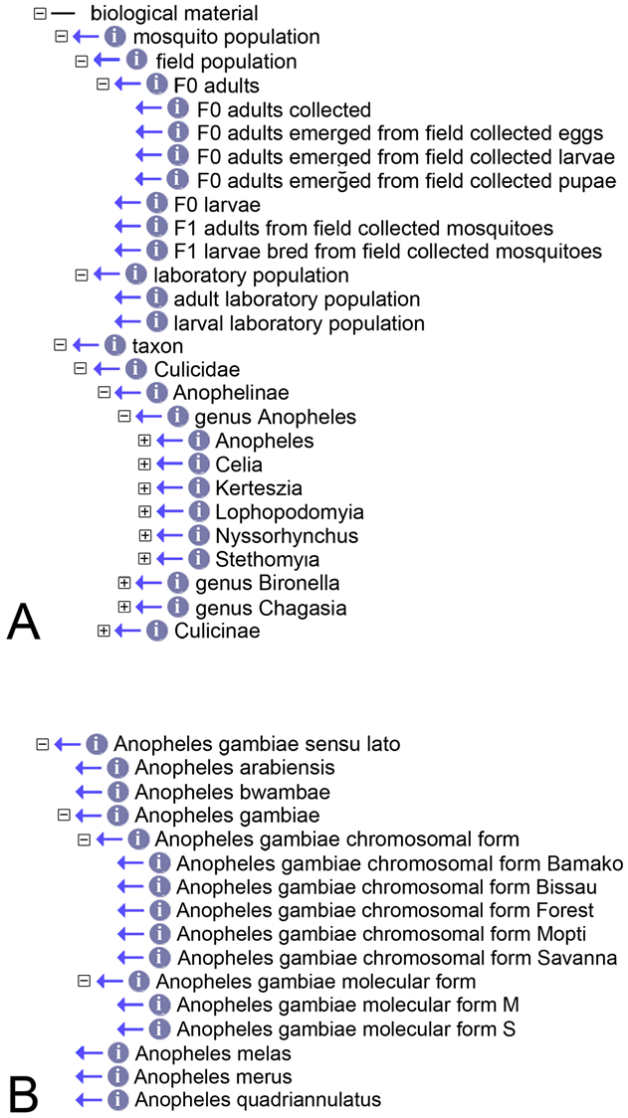
The “*biological material*” class. The figure shows, in a depth of 5 levels, the class “biological material” (A) and, within the “*Cellia*” subgenus, the *Anopheles gambiae* s.s.-related terms (B), that also include the chromosomal and molecular forms.

This class, the largest one in the MIRO with 3,790 terms, describes all parameters that define the mosquito populations investigated (Figure 2). Its two main nodes are self-explanatory, one defining details of the population under study, including the biological stages of the individual specimens collected and sampled, as well as the kind of populations studied (field or established laboratory stock), while the other eventually defining the species under study (Figure 2A). As mentioned above, we have restricted the taxa listed in the MIRO to mosquitoes as these represent the main vectors of disease (e.g. Dengue, filariasis, malaria, yellow fever, etc.). We will eventually restrict the species of mosquitoes listed in the ontology to those that have already been described as actual vectors, and expand the ontology to cover non-mosquito vector arthropods (e.g. ticks, sand flies, etc.). For the present compilation of the different mosquito taxa we used primarily the Systematic Catalog of the *Culicidae* found at the Walter Reed Biosystematics Unit (WRBU, http://www.mosquitocatalog.org/species/taxonomy.asp). All taxa were linked to their parents, *i.e.* to the respective subgenus and genus, by *is_a* relationships and all synonyms listed in the WRBU catalogue were also registered in the ontology. We have also gone beyond the WRBU catalogue by including in the MIRO cryptic species such as incipient species “*sensu* Coluzzi” or chromosomal and molecular forms [30]. This obviously means that at present the S and M forms of *An. gambiae* s.s., for which extensive studies are being conducted, can equally be found in the ontology, and a particular analysis can be annotated accordingly (Figure 2B). Should future entomological research make it necessary to include similar data for other insect species groups the ontology can naturally be expanded in this respect.

#### Insecticidal substance

**Figure 3:**
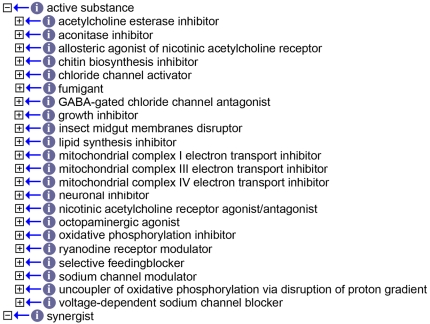
The groups of insecticides in MIRO. The figure shows the list of the groups of substances with differing modes of action containing the individual insecticides.

Two “catalogues” are available for the construction of the sub-ontology defining insecticides. These are ChEBI an open ontology of Chemical Entities of Biological Importance [31] and the IRAC catalogue (http://www.irac-online.org/eClassification/) a structured vocabulary compiled by the Insecticide Resistance Action Committee (http://www.irac-online.org/). Upon our request, the ChEBI group included in their ontology all insecticides listed by IRAC, and it now represents an optimal controlled vocabulary for those substances that could be used in the MIRO. Nevertheless, the IRAC eClassification list has the advantage of being immediately recognized and accepted by the IR (Insecticide Resistance) community as standard reference. Its structure is based on the mode of action of the individual insecticides but, to some extent, it is rather problematic on the level of an ontology. For example, it is based on a fairly rigid classification that, among others, leads to nameless or “non-existent” classes, or to classes that are not definable on either chemical or functional level (e.g. a class of compounds of “unknown mode of action” or a class of “miscellaneous non-specific inhibitors” [*sic*]. Nevertheless, given 1) the familiarity of the IR community with this classification and 2) the fact that the MIRO is meant to be an application ontology, we decided to use this, after some reorganization, as the primary scaffold for the construction of the ontology (Figure 3). All insecticides were, nevertheless, cross-referenced to ChEBI, as this ontology already represents the key ontology that links biology to chemical substances. The reason for cross-referencing, rather than using the ChEBI ID codes is to be found in the fact that in the MIRO all insecticides are defined, which is not the case for ChEBI. A total of 22 different modes of action were retained for the classification of insecticides developed and included in the IRAC list effective December 2008. Finally we also included in the ontology a class containing synergists; two pertinent groups of synergists were listed in previous versions of the IRAC eClassification, but are no longer present. In spite of this, we incorporated them in the MIRO, given their potential significance in the actual usage of insecticides. The “*Insecticidal substance*” class now contains 287 terms across all groups of substances.

#### Resistance

**Figure 4:**
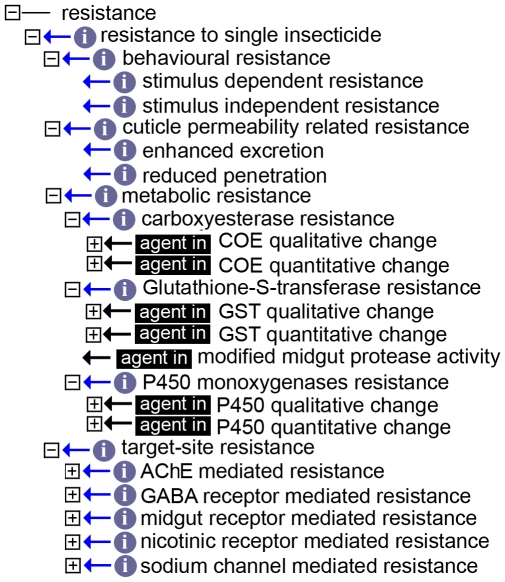
The “*resistance*” class. The “*resistance*” class has been opened to show the different contents. The black boxes denote an “*agent_in*” relation.

This class (73 terms), somewhat hindered by the jargon used by the IR community, lists all mechanisms known at this time. The four main categories used are behavioral and metabolic resistance, resistance due to changes in the permeability of the insect's cuticle and, finally, target-site resistance (Figure 4). Both “*behavioral resistance*” and “*cuticle permeability related resistance*” only list two self-explanatory children each: “*stimulus-dependent*” and “*stimulus-independent*” for the former, and “*enhanced excretion*” and “*reduced penetration*” for the latter. In contrast, the remaining two classes are more complex. The “*metabolic resistance*” class includes different facets of resistance connected to qualitative and quantitative changes of the activity of carboxyesterases (COE) and glutathione S-transferases (GST) and P450 monoxygenases. Furthermore a single child describes resistance due to modified midgut protease activity, *i.e.* processes related to the usage of biological insecticides such as the ones derived from *Bacillus thuringensis* or *B. sphaericus*. Finally target site resistance deals with known described mutations of specific genes.

#### Method

The final class was to some extent a problematic one. The reason for this was not the actual ontology construction but, rather, a problem of orthogonality. This class covers most, if not all methods that are directly used for the analysis of insecticide resistance in a mosquito population (a total of 137 terms). The methods vary from catch methods for field populations to molecular biological techniques (see Figure 1). While the former are straightforward and relatively easy to catalogue the latter pose certain dilemmas. These range from the terms used as such (e.g. “*bioassay*” or “*biochemical assay*” which may be too general) to the question of whether terms such as “*real-time PCR*” or “*RT-PCR*” that are outside the “narrow” field of insecticide resistance should be included. We decided eventually to include all techniques that are routinely used for the analysis of insecticide resistance. The choice was made based on the fact that the ontology OBI (personal communication, the OBI Consortium http://purl.obofoundry.org/obo/obi), which is currently being developed and which will describe life science and clinical investigations, is far from completion, and the MIRO would be missing a crucial component if we were to exclude the relevant terms. Similar limitations existed for the techniques used for species identification and here we decided to keep the terms general without going into details. The species identification part is short and only describes the seven most common ways of identifying individual mosquito species. These include classical procedures (chromosomal banding patterns, cross mating experiments, morphology, and salinity tolerance tests) as well as biochemical (isozyme electrophoresis) and molecular (DNA probes, PCR). Of course, like is the case for all components of the MIRO, the ontology can be expanded or changed accordingly in the future if changes are deemed necessary.

### The IRbase database

**Figure 5:**
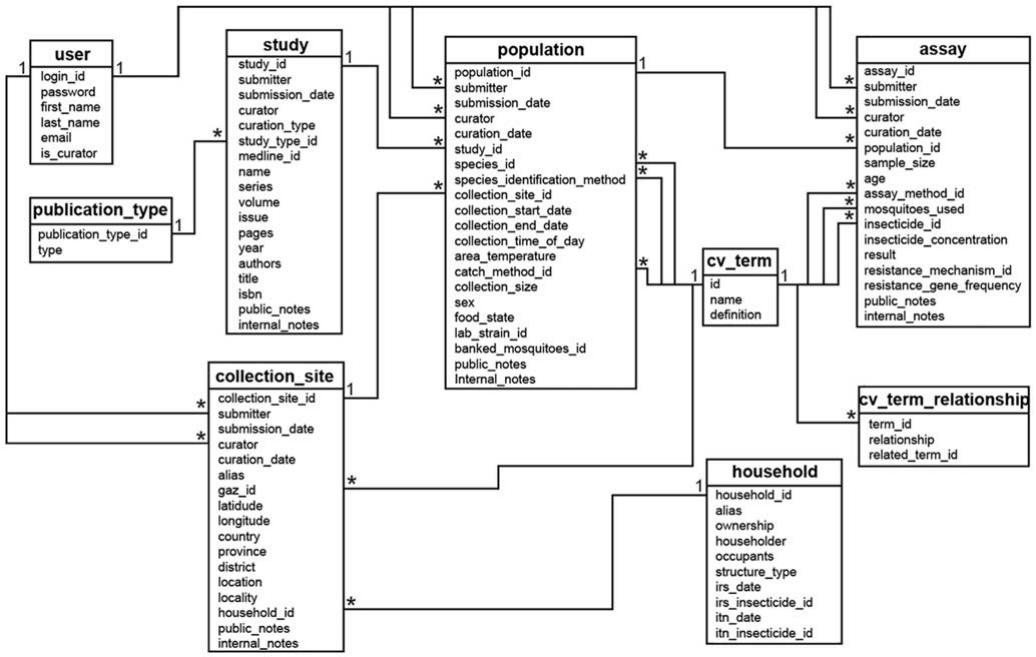
The IRbase schema. The figure shows the different tables that make up the schema. 1 and * denote a “one to many” relationship.

Based on feedback from the malaria entomology research community it was decided several years ago to include in AnoBase, the *Anopheles* database [21], a section on insecticide resistance; this tool was later transferred to and included in VectorBase [23],[24] after this comprehensive genome database was established. The section consisted only of a series of manually-curated, already published studies; its role, therefore, was mostly to make data available to the community in a fashion that would be independent of the need for a library, rather than a use as an on-line epidemiological tool. The new IRbase in contrast is meant to serve as an expanding repository of associated data, which can be searched in a detailed fashion, thus providing immediately applicable information. Furthermore, IRbase now covers vectors of more diseases than the previous database that was only restricted to malaria. These are the reasons for designing a relational schema *de novo* (see Figure 5). It was our intention to design a schema that would easily enable both the addition of novel tables and the incorporation of IRbase into a larger and more complicated entity, which could be expanded later to encompass additional items linked to the control of vector-borne diseases.

The nine distinct tables can be distinguished in two major categories: While two of them (cv_term) handles all MIRO terms, including GAZ, and their relationships, the remaining are there to handle, mostly, ontology-independent items. These include, most prominently, description of the study in terms of details of the collection site, the mosquito population sampled (including collection dates, etc.) and the assay(s) performed. The “household” table is presently not in use by IRbase, but it has been included by request as it could be needed by decision support systems currently under development for Dengue and malaria [32]. The schema allows for a high degree of interoperability due to the enhanced usage of the ontology component, and it enhances the two distinctive features of IRbase, *i.e.* the two interactive components, *search* and *curator's tool*, both of which are accessible through a simplified web interface.

In addition to the completely new architecture of the database and to the fact that the software used is free and open source, IRbase has some key characteristic features: i) The data are stored in the database using MIRO terms wherever possible; ii) the Gaz geographic ontology is used for storing location data and the output can be viewed using maps; iii) extensive use of Ajax (Previously AJAX: Asynchonous JAvascript XML) is made in order to minimize network traffic and improve look and feel [33]. Moreover IRbase was built around basic entities:

1. “S*tudy data*” - a storage space for the data pertaining to an individual “study”. The “study” could be an entire study, previously published or not, on an entire population or parts thereof, pertaining to one or more insecticides; the pertinent data would include the “owner” of the particular data, time it was carried out, the publication record when available, etc.

2. “*Collection site*” - common names of the collection site(s), their alias(es) and, most importantly, the geographic coordinates. Should these not be available through the submitter of the data, the IRbase curators will assign values based on available information and feedback. For those names that already exist in Gaz the Gaz ID is also stored.

The alias is an ID that the submitter can use for faster data entry: the collection site needs to be defined once and from thereon the alias can be used to identify that particular site. Collection sites that have no Gaz ID are exported and sent to the curators of that ontology for ID assignment.

3. “*Insect collections*” - this area holds information such as the species name, the collection date, the catch method, the sex, food state etc. of the specimens (field collected or lab bred) that were subsequently used to test resistance.

4. “*Assay data*” - The actual data expressing the findings and referring to the methods used, the conditions (insecticide concentration) and the results of an assay, etc.

#### The user interface

A brief manual is presented along with the search forms. There are two ways for entering search parameters into them. The first one is to use the drop-down menus and find the requested term by following the correct path. This obviously implies that the user is familiar with the MIRO or has a good knowledge for some of the properties of the requested term. For example, to find the insecticide “deltamethrin”, the user must know that this insecticide belongs to the pyrethroid family and that this family of insecticides modulates sodium channels. Alternatively, auto-complete input boxes can be used. In these boxes the user needs to type two letters from the requested term and a list of all possible matching terms will appear. As more letters are typed the search is narrowed down to the decreasing number of options in the list. When the requested term appears on the list it can be clicked on and it will now appear in the input box. All terms are listed alphabetically in both the drop-down and the autocomplete menus.

Search criteria include species sites, year of collection, pertinent insecticide resistance mechanism, assay method, mosquitoes used, catch method and more. One criterion only may be used, or any combination of two or more of the above criteria. With the exception of the year of collection all remaining search criteria are ontology-based searches. As a result of this, the search algorithm implemented will also search for all the descendant terms of the term specified, and therefore searches can be narrowed in the process. Returned data are presented in descending chronological order, regardless of whether the collection year was set as a search criterion or not.

Users who want to utilize IRbase's data to run their own tests can set their export criteria and obtain the relevant data in a tab separated values (tsv) text file. This file can be opened in any spreadsheet application or be imported into a database.

#### Maps

**Figure 6:**
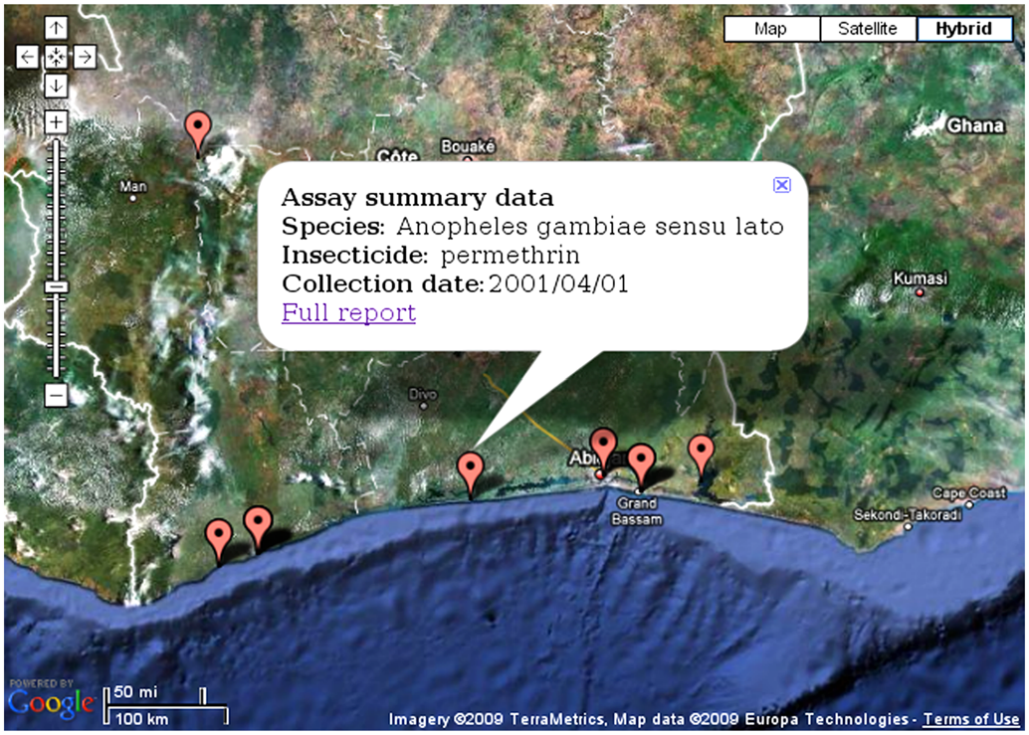
A map output providing summary information. The figure shows a screen shot of a search involving studies in Cote d'Ivoire. The pink “droplets” point to the sites of mosquito collections while the balloon that appears when clicking on any one of them includes summary data for the collection site. A link to the respective full report is also provided in the balloon.

In addition to the text-based interface to view data, IRbase also provides a map-based interface to access the same data (Figure 6). This interface utilizes Google Maps to visualize the data and is very rich in features such as grouping by color, zooming in and out, adding layers of related data, etc. By clicking at the collection sites marked on the map, a pop up balloon will appear with the same data, but also with a link to a detailed report (Figure 3). The map tool fully depends on the availability of geographical coordinates. As some of the older data are not linked to such information, this will have to be supplied manually by the IRbase curators before these studies can be incorporated. When the page is first loaded, a world map with all the collection sites spotted with small markers will appear. After leaving the mouse pointer on one of these markers, one line of text will appear displaying some of the information regarding the particular collection site such as species name, collection dates, and insecticide used. We stress here that the map section is continuously being improved in order to provide the users with a “friendlier” tool.

#### Data input

Data can be submitted to IRbase either online, via a web interface, or offline, using a spreadsheet template. These tools are available to the community upon request. Similarly a streamlined edition of the user and submitter/curator interface as well as the database can be loaded onto a laptop for offline data entry. This offers the advantage of a “limited” usage of IRbase even under conditions of limited access to the Internet (e.g. field trips). Again, users wanting to take advantage of this facility can contact VectorBase in order to obtain a user name and password.

### Conclusion

We described here a set of IT tools to be used for the analysis of insecticide resistance in wild populations of insect disease vectors and in particular mosquitoes. The concept of intimately linking a dedicated database to a specific application ontology describing the field offers the advantage that the database can later be easily expanded to include additional items and offer further tools. This fact, in our case, can form the overall foundation or one of the pillars of a comprehensive tool, which could be used to globally monitor insecticide resistance; this would form the basis for a global decision support system for malaria and/or other vector-borne diseases.

A database on insecticide resistance, the Arthropod Pesticide Resistance Database (APRD), can already be found in the world wide web (http://www.pesticideresistance.org/). APRD covers a large variety of arthropods, but its philosophy is different from the one of IRbase. It provides reports of instances of occurrence of resistance, without any precision as to the exact location and the actual data. Although useful as a general indicator of resistance, especially in the domain of agriculture, the lack of geographic accuracy, combined with the lack of a map interface makes this database less suitable as a tool that could be used either by itself, or in combination to a modern, IT-based decision support system.

Such decision support systems are considered to be a prerequisite for the efficient control of insect vector populations. Many potential components of such systems have been described (see for example [32], [34]–[35]), especially components that are based on GIS. Our tool has for the moment the capacity to depict data of insecticide resistance on a map provided the geographic coordinates have been incorporated in the data collection. Since many of the data that will populate IRbase are old, some of the coordinates will have to be input manually; once this has been the case, it will be possible to link all available information to maps based on, and retrieved from Google Earth.

The MIRO/IRbase set of tools is presently focused completely on insecticide resistance linked to mosquitoes of medical importance. The open source policy linked to the MIRO, an ontology that abides with the OBO Foundry rules, makes it easy to further develop these tools in order to later include data of agricultural interest as well, should an interested party turn up. In that sense one should also consider the fact that development of resistance detected in disease vectors can often be traced back to the often-improper use of insecticides in agriculture (see [36] for a discussion of that problem).

We are currently in the process of populating IRbase with both data from the literature and data that are being collected from the field. This is done in collaboration with the international community in the frame of large consortia (e.g. African Network on Vector Research, Innovative Vector Control Consortium, WHO/Gates Foundation Vector Biology and Control Project, etc.), as well as on the basis of smaller individual research networks. We hope that, this way, IRbase will soon be established as the global repository for data insecticide resistance.
